# An Updated Meta-Analysis: Risk Conferred by Glutathione S-Transferases (*GSTM1* and *GSTT1*) Polymorphisms to Age-Related Cataract

**DOI:** 10.1155/2015/103950

**Published:** 2015-01-27

**Authors:** Rong-feng Liao, Min-jie Ye, Cai-yuan Liu, Dong-qing Ye

**Affiliations:** ^1^Department of Epidemiology and Biostatistics, School of Public Health, Anhui Medical University, 81 Meishan Road, Hefei, Anhui 230032, China; ^2^Department of Ophthalmology, The First Affiliated Hospital of Anhui Medical University, Hefei, 218 Jixi Road, Hefei, Anhui 230022, China; ^3^Department of Ophthalmology, The Hospital of University of Science and Technology of China, Jinzhai Road, Hefei, Anhui 230026, China

## Abstract

*Purpose*. To study the effects of glutathione S-transferase M1 (GSTM1) and T1 (GSTT1) polymorphisms on age-related cataract (ARC). *Methods*. After a systematic literature search, all relevant studies evaluating the association between GSTs polymorphisms and ARC were included. *Results*. Fifteen studies on GSTM1 and nine studies on GSTT1 were included in this meta-analysis. In the pooled analysis, a significant association between null genotype of GSTT1 and ARC was found (OR = 1.229, 95% CI = 1.057–1.429, and *P* = 0.007). In subgroup analysis, the association between cortical cataract (CC) and GSTM1 null genotype was statistically significant (OR = 0.713, 95% CI = 0.598–0.850, and *P* < 0.001). In addition, GSTM1 null genotype was significantly associated with ARC causing risk to individuals working indoors and not individuals working outdoors. The association between GSTT1 null genotype and risk of ARC was statistically significant in Asians (OR = 1.442, 95% CI = 1.137–1.830, and *P* = 0.003) but not in Caucasians. *Conclusions*. GSTM1 positive genotype is associated with increased risk of CC and loses the protective role in persons who work outdoors. Considering the ethnic variation, GSTT1 null genotype is found to be associated with increased risk of ARC in Asians but not in Caucasians.

## 1. Introduction

Cataract is one of the most common causes of visual impairment and blindness all over the world. 80% of cataract is age-related cataract (ARC), which is classified as cortical cataract (CC), nuclear cataract (NC), or posterior subcapsular cataract (PSC), according to the location of the opacity in the lens [1, 2]. Although the pathogenesis of ARC is not fully understood, many epidemiologic studies have noted that oxidative stress and genetic factors may play major roles in the development of ARC [3].

There are many cellular defense mechanisms that protect the human lens from oxidative damage. The glutathione S-transferase (GST) is one of the detoxification enzyme systems and plays important role in inactivating endogenous and exogenous toxic products under oxidative stress. The GST isoenzymes have been reported to express classes, mu, theta, and pi, in human lens tissue [4–7].* GSTM1*/*T1* polymorphisms are the most common polymorphisms of GST enzymes, and they have been associated with many diseases, such as rheumatoid arthritis, age-related macular degeneration, oral leukoplakia, prostate cancer, lung cancer, and cervical neoplasia [8–13].

Possible association between the* GSTM1/T1* polymorphisms and ARC has been investigated in many studies however with conflicting results. Recently, a meta-analysis was performed to evaluate the association between* GSTM1*/*T1* polymorphisms and ARC [14]. Ever since, new studies of* GSTM1/T1* polymorphisms in cataract have been published, shedding new light on the topic. We performed an updated meta-analysis of the available studies to better ascertain the association of* GSTM1/T1* polymorphisms and the risk of ARC.

## 2. Materials and Methods

### 2.1. Literature Search Strategy

We conducted a comprehensive systematic search to identify relevant studies from Medline, Embase, PubMed, Web of Science, and China National Knowledge Infrastructure using keyword combinations: “glutathione S-transferase or* GST* or* GSTM1* or* GSTT1*” and “cataract or age-related cataract or senile cataract or ARC.” When there was more than one publication using the same patient sample, only the one with the largest sample size was selected.

### 2.2. Inclusion Criteria and Data Extraction

Studies that met all the following criteria were regarded eligible: (1) case-control study, (2) investigation of the association between* GSTM1/T1* polymorphisms and ARC, and (3) providing the information on genotype frequencies of* GSTM1/T1* polymorphism in both cases and controls. We collected the following information from each study as initial data: the first author's name, publication year, ethnicity (country), and the number of* GSTM1* and* GSTT1* genotypes in the cases and controls. The articles were reviewed independently by two investigators (Minjie Ye and Caiyuan Liu), who also extracted data. The quality of studies was also evaluated based on the STROBE quality score systems [15]. A third reviewer (Rongfeng Liao) served as the third reviewer if there was any disagreement.

### 2.3. Statistical Analysis

The association between* GSTM1/T1* polymorphisms and risk of ARC was expressed as odds ratio (OR) and 95% confidence interval (CI). The statistical analysis was performed using Stata 11.0 (StataCorp, College Station, TX). An I^2^ statistic was conducted to evaluate whether inconsistencies among studies were attributed to heterogeneity rather than chance. When there was no heterogeneity of the results of the publications, we used the fixed effects model (Mantel-Haenszel method) [16]. Otherwise, we used the random effects model (DerSimonian-Laird method) [17]. Subgroup analyses were performed on the basis of ethnicity, gender, ARC subtypes, and the work place of the study subjects (indoor and outdoor work place). Finally, the Egger weighted regression method and funnel plots were used to evaluate publication bias visually.

## 3. Results

### 3.1. Characteristics of Studies

**Figure 1:**
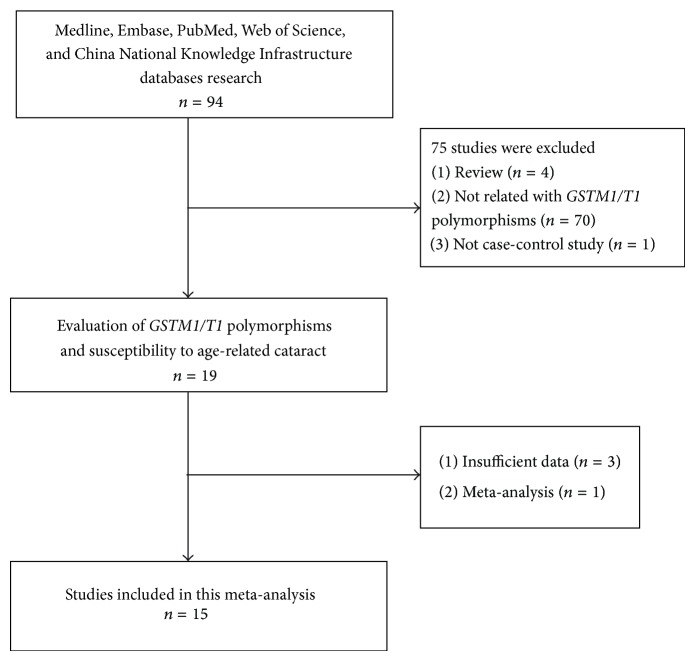
Flow chart showing study selection procedure.

Flow diagram of studies included in this meta-analysis is provided in Figure 1. Fifteen studies [18–32] were included in the meta-analysis of the* GSTM1* genotype (3165 cases, 2105 controls), and nine studies were included in the meta-analysis of* GSTT1* (2374 cases, 1544 controls). For the meta-analysis of* GSTM1*, six studies on Asians and nine on Caucasians were included. While for the analysis of* GSTT1*, two studies on Asians and seven studies on Caucasians were included. The characteristics of the studies included in the meta-analysis are presented in Table 1.

### 3.2. Meta-Analysis Results

**Figure 2:**
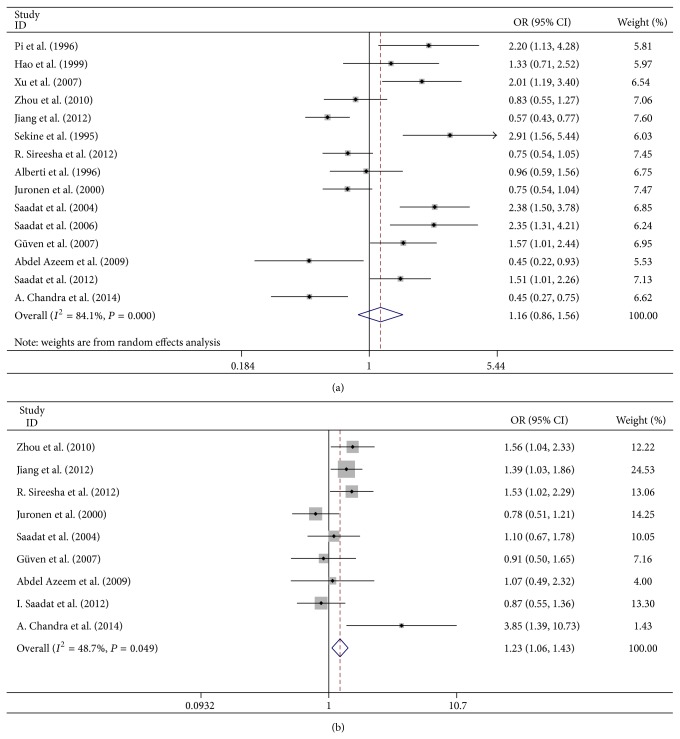
Forest plot of the association between* GSTM1*/*T1* null genotype and age-related cataract (ARC). (a) Forest plot of the association between* GSTM1* null genotype and ARC. (b) Forest plot of the association between* GSTT1* null genotype and ARC.

The forest plot of the* GSTM1* and* GSTT1* genotypes is shown in Figures 2(a) and 2(b), respectively. No association was detected between* GSTM1* null genotype and ARC in the overall analysis (OR = 1.161, 95% CI = 0.863–1.563, and *P* = 0.324). The association between* GSTT1* null genotype and risk of ARC was statistically significant (OR = 1.229, 95% CI = 1.057–1.429, and *P* = 0.007).

**Figure 3:**
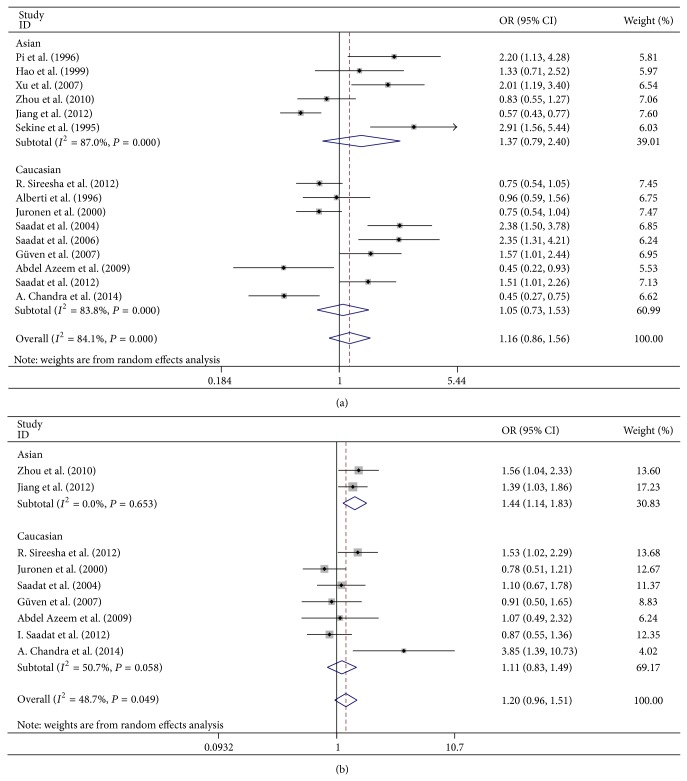
Ethnicity-based subgroup analysis of the correlation between* GSTM1/T1* null genotype and age-related cataract (ARC). (a) Ethnicity-based subgroup analysis of the association between* GSTM1* null genotype and ARC. (b) Ethnicity-based subgroup analysis of the association between* GSTT1* null genotype and ARC.

**Figure 4:**
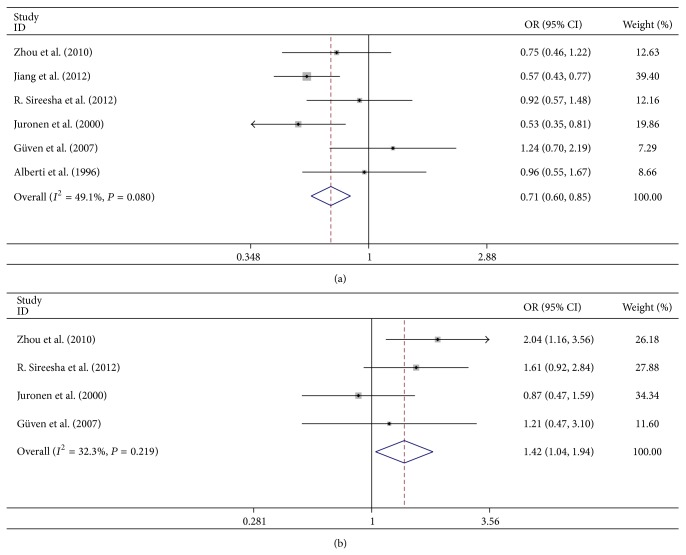
Subgroup analysis of the correlation between* GSTM1*/*T1* null genotype and subtypes of age-related cataract (ARC). (a) Subgroup analysis of the association between* GSTM1* null genotype and cortical cataract (CC). (b) Subgroup analysis of the association between* GSTT1* null genotype and posterior subcapsular cataract (PSC).

**Figure 5:**
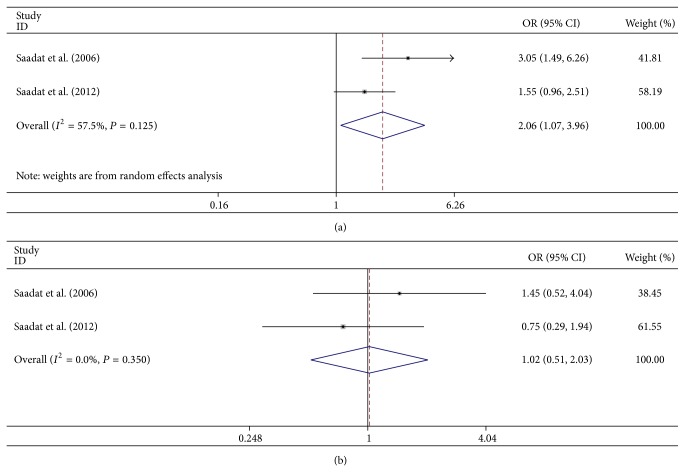
Working place-based subgroup analysis of the correlation between* GSTM1/T1* null genotype and age-related cataract (ARC). (a) The association between* GSTM1* null genotype and ARC in indoor subjects. (b) The association between* GSTM1* null genotype and ARC in outdoor subjects.

Subgroup analyses on ethnicity indicated that the association between* GSTM1* null genotype and risk of ARC was not significant in Asians or Caucasians (OR = 1.372, 95% CI = 0.786–2.396, and *P* = 0.266; OR = 1.053, 95% CI = 0.726–1.526, and *P* = 0.785, Figure 3(a)). The association between* GSTT1* null genotype and risk of ARC was statistically significant in Asians but not in Caucasians (OR = 1.442, 95% CI = 1.137–1.830, and *P* = 0.003; OR = 1.113, 95% CI = 0.830–1.492, and *P* = 0.474, resp., Figure 3(b)). In subgroup analyses, by gender, we found that* GSTM1* null genotype was not associated with ARC in female or male group (OR = 1.016, 95% CI = 0.444–2.324, and *P* = 0.970; OR = 0.892, 95% CI = 0.582–1.365, and *P* = 0.598, resp.). Similar results were found for the association between* GSTT1* null genotype and risk of ARC in Asian female or male group (OR = 1.281, 95% CI = 0.972–1.687, and *P* = 0.078; OR = 1.288, 95% CI = 0.977–1.698, and *P* = 0.073, resp.). When analyzed by subtypes of ARC, the* GSTM1* null genotype was significantly correlated with CC (OR = 0.713, 95% CI = 0.598–0.850, and *P* < 0.001; Figure 4(a)) but not with NC, PSC, or mixed type (MT) (OR = 0.887, 95% CI = 0.685–1.148, and *P* = 0.363; OR = 1.042, 95% CI = 0.797–1.362, and *P* = 0.765; OR = 0.937, 95% CI = 0.510–1.722, and *P* = 0.834, resp.).* GSTT1* null genotype was significantly correlated with PSC (OR = 1.421, 95% CI = 1.043–1.936, and *P* = 0.026; Figure 4(b)) and marginally correlated with CC (OR = 1.226, 95% CI = 0.999–1.504, and *P* = 0.051). However, there were no significant associations between* GSTT1* null genotype and NC or MT (OR = 0.921, 95% CI = 0.524–1.617, and *P* = 0.774; OR = 1.209, 95% CI = 0.663–2.204, and *P* = 0.535, resp.). In subgroup analyses, by the work place, we found that the association between* GSTM1* null genotype and ARC was statistically significant in the indoor subjects but not in the outdoor subjects (OR = 2.062, 95% CI = 1.074–3.961, and *P* = 0.030, Figure 5(a); OR = 1.019, 95% CI = 0.511–2.034, and *P* = 0.957, Figure 5(b)).

To investigate the association between profiles of* GST* genotypes and the risk of ARC, we examined the association between combination of* GSTM1* null and* GSTT1* null genotypes and risk of ARC but failed to detect any association between them in all populations (OR = 1.069, 95% CI = 0.843–1.356, and *P* = 0.581). Similarly, the combination of GSTM1 positive and GSTT1 positive/GSTM1 positive and GSTT1 null genotypes was not associated with ARC risk (OR = 1.005, 95% CI = 0.658–1.536, and *P* = 0.981; OR = 1.281, 95% CI = 0.840–1.954, and *P* = 0.250, resp.). The results of subgroup analyses are presented in Table 2.

### 3.3. Potential Publication Bias

**Figure 6:**
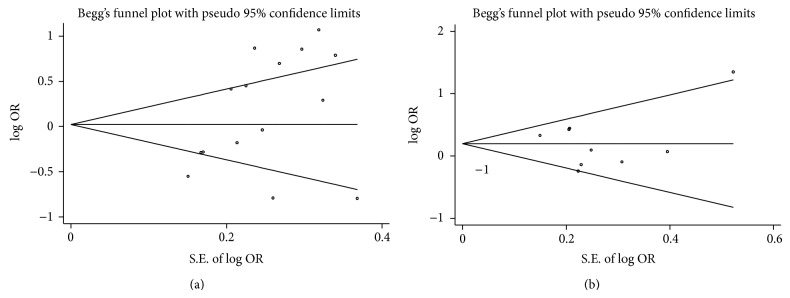
Begg's funnel plots of publication bias analyses. (a) Funnel plot of* GSTM1* polymorphism and risk of ARC. (b) Funnel plot of* GSTT1* polymorphism and risk of ARC.

Funnel plots and Egger's test were generated to evaluate potential publication bias for* GSTM1* (Figure 6(a)) and* GSTT1* (Figure 6(b)). A statistically significant publication bias was detected for* GSTM1* (Egger's test, *P* = 0.048), but no publication bias was detected for* GSTT1* (Egger's test, *P* = 0.908).

## 4. Discussion

Causality of age-related cataract is considered to be multifactorial, and oxidative stress and genetic factors are considered the major factors in its development. It has been noted that* GST* polymorphisms act as genetic risk factor for ARC. However, results of the studies examining the association between* GSTM1/T1* polymorphisms and ARC have been inconsistent. A meta-analysis performed by Sun et al. in 2010 reported that* GSTM1* and* GSTT1* null genotypes were associated with increased risk of ARC in Asians but not in Caucasians [14]. Thereafter, several additional clinical studies that evaluated the association of* GSTM1/T1* polymorphisms and ARC have been reported. We therefore updated the present meta-analysis which included a larger sample size to provide a more reliable association between* GSTM1/T1* polymorphisms and ARC susceptibility.

Compared to Sun's study, our study has some particular strength. First, we added four studies with large samples size, the absence of which might lead to a deviation in the results of Sun's study. Second, given that GSTs play a vital role in detoxification of xenobiotics and protection of lens from the oxidative damage, we performed subgroup analysis based on the work place (outdoor/indoor) to investigate the possible contribution of* GSTM1/T1* polymorphisms to susceptibility to ARC. Third, it has been noted that combination of the GST polymorphisms rather than individual polymorphism makes persons more susceptible to genotoxic insults [33]. Considering the possible additive effect of different* GST* genotypes, the association between the genotype profile and ARC risk was also estimated.

Inconsistent with the previous meta-analysis, our finding indicated that the* GSTM1* null genotype was not associated with the ARC risk in Asian populations. Interestingly, the result of subgroup analyses based on ethnic illustrated that the* GSTM1* null genotype was associated with decreased CC risk (*P* < 0.001). Meanwhile, in subgroup analysis by the work place, we found that* GSTM1* positive genotype was not associated with decreased risk of ARC in outdoor subjects. Why does the* GSTM1* positive genotype increase the CC risk and lose its protective role in individuals who were occupationally exposed to sunlight? The following reasons may account for the results. (1) Despite the fact that GST enzymes are important in defense against oxidative stress, they also participate in reactions that create toxic products which may result in structural alterations to the proteins and then cause lens opacification [34]. (2) The activity of GST is significantly decreased in cataractous lens compared with that in normal lens, and hence the positive genotype of* GSTM1* may lose its ability to prevent cataract development. (3) Absence of the protection of* GSTM1* enzyme may stimulate other cellular defense mechanisms to detoxify the substrates. (4) It has been reported that UVB (ultraviolet radiation b) irradiation results in inhibitory effect on GST activity in the skin [35]. Therefore, this might suggest that activity of GST is inhibited in the human lens after UVB irradiation.

In our study,* GSTT1* null genotype was associated with increased risk of ARC in the Asians but not in the Caucasians, which may be due to the difference between ethnic and the distributions of* GSTT1* null genotype. It has been reported that the frequency of the* GSTT1* null genotype is higher in Asian population compared with other populations [36]. The frequency of* GSTT1* null genotype is nearly 50% in both the Chinese and Japanese populations [37–40]. Nevertheless, the Caucasian population has a lower frequency (11.0%–37.9%) [41, 42]. Thus, different ethnic populations may have different susceptibility to ARC depending on the pattern of* GSTT1* gene polymorphism. This could partly explain why the* GSTT1* null genotype is associated with increased risk of ARC in the Asian population. In subgroup analyses stratified by subtypes of ARC, we also found that the* GSTT1* null genotype increased the risk of CC and PSC.

Considering the role of* GSTM1* positive and* GSTT1* null genotype in ARC development, investigation of the association between the combination of* GSTM1* positive and* GSTT1* null genotypes and ARC risk should be suggested. Therefore, we did this analysis and found the combination of* GSTM1* and* GSTT1* null genotypes was associated with 1.281-fold increased risk of ARC, although the association was not significant.

Gender differences have also been observed on the association between* GST* polymorphisms and human skin and colon mucosa [43, 44]. Thus, we performed subgroup analysis on the basis of gender. Consistent with Sun's findings, our results illustrated that gender had no effect on the association between* GSTM1/T1* polymorphisms and ARC.

Despite the fact that we made an accurate and comprehensive analysis, limitations still existed in our study. First, our meta-analysis only included studies with accessible full-text articles, in English or Chinese. Therefore, the absence of some otherwise eligible studies that were unpublished or reported in other languages could lead to some inevitable publication bias. Second, due to the lack of detailed data, subgroup analysis stratified by habits like smoking and alcohol consumption was not conducted. Third, the type and degree of opacification were classified using the lens opacities classification system II (LOCS II) or lens opacities classification system III (LOCS III). Difference of classified methods among the studies might have affected the results.

In summary, this study suggested that* GSTM1* positive genotype is associated with increased risk of CC and loses the protective role in persons who work outdoors.* GSTT1* null genotype confers increased risk of ARC in Asians but not in Caucasians.

## Figures and Tables

**Table 1:** Characteristics of studies included in the meta-analysis.

First author	Year	Ethnicity (country)	Sample size		Number of null genotypes		References	Quality score
			Cases	Controls	Cases	Controls		
*GSTM1*:								
Pi	1996	Asian (China)	59	112	41	57	[18]	18
Hao	1999	Asian (China)	77	76	41	35	[19]	17
Xu	2007	Asian (China)	120	118	81	60	[20]	19
Zhou	2010	Asian (China)	279	145	171	95	[21]	29
Jiang	2012	Asian (China)	422	312	176	173	[22]	31
Sekine	1995	Asian (Japan)	138	62	101	30	[23]	20
Sireesha	2012	Caucasian (India)	455	205	177	94	[24]	32
Alberti	1996	Caucasian (Italy)	202	98	99	49	[25]	23
Juronen	2000	Caucasian (Estonia)	503	202	240	111	[26]	25
Saadat	2004	Caucasian (Iran)	150	150	90	58	[27]	25
Saadat	2006	Caucasian (Iran)	95	95	56	36	[28]	23
Güven	2007	Caucasian (Turkey)	195	136	105	58	[29]	29
Abdel Azeem	2009	Caucasian (Egypt)	53	73	23	46	[30]	22
Saadat	2012	Caucasian (Iran)	186	195	104	89	[31]	26
Chandra	2014	Caucasian (India)	131	126	43	68	[32]	25
*GSTT1*:								
Zhou	2010	Asian (China)	279	145	146	60	[21]	29
Jiang	2012	Asian (China)	422	312	221	138	[22]	31
Sireesha	2012	Caucasian (India)	455	205	123	40	[24]	32
Juronen	2000	Caucasian (Estonia)	503	202	73	36	[26]	25
Saadat	2004	Caucasian (Iran)	150	150	49	46	[27]	25
Güven	2007	Caucasian (Turkey)	195	136	29	22	[29]	29
Abdel Azeem	2009	Caucasian (Egypt)	53	73	16	21	[30]	22
Saadat	2012	Caucasian (Iran)	186	195	49	57	[31]	26
Chandra	2014	Caucasian (India)	131	126	18	5	[32]	25

*GSTM1*: glutathione S-transferase M1; *GSTT1*: glutathione S-transferase T1.

**Table 2:** Subgroup analysis of the association between *GSTM1* and *GSTT1* polymorphisms and the risk of age-related cataract.

Groups	Number of studies	Statistical method	OR (95% CI)	*P*	References
*GSTM1*:					
All studies	15	Random	1.161 (0.863–1.563)	0.324	[18–32]
Ethnicity:					
Asian	6	Random	1.372 (0.786–2.396)	0.266	[18–23]
Caucasian	9	Random	1.053 (0.726–1.526)	0.158	[24–32]
Gender:					
Female	5	Random	1.016 (0.444–2.324)	0.970	[22, 24, 27, 29, 30]
Male	5	Random	0.892 (0.582–1.365)	0.598	[22, 24, 27, 29, 30]
Subtype:					
CC	6	Fixed	0.713 (0.598–0.850)	**<0.001**	[21, 22, 24–26, 29]
NC	5	Fixed	0.887 (0.685–1.148)	0.363	[21, 24–26, 29]
PSC	4	Fixed	1.042 (0.797–1.362)	0.765	[21, 24–26, 29]
MT	3	Random	0.937 (0.510–1.722)	0.834	[24, 26, 29]
Environmental risk factors:					
Outdoor	2	Fixed	1.019 (0.511–2.034)	0.957	[28, 31]
Indoor	2	Random	2.062 (1.074–3.961)	**0.030**	[28, 31]
*GSTT1*:					
All studies	9	Fixed	1.229 (1.057–1.429)	**0.007**	[21, 22, 24, 26, 27, 29–32]
Ethnicity:					
Asian	2	Fixed	1.442 (1.137–1.830)	**0.003**	[21, 22]
Caucasian	7	Fixed	1.113 (0.830–1.492)	0.474	[24, 26, 27, 29–32]
Gender:					
Female	5	Fixed	1.281 (0.972–1.687)	0.078	[22, 24, 27, 29, 30]
Male	5	Fixed	1.288 (0.977–1.698)	0.073	[22, 24, 27, 29, 30]
Subtype:					
CC	5	Fixed	1.226 (0.999–1.504)	0.051	[21, 22, 24, 26, 29]
NC	4	Random	0.921 (0.524–1.617)	0.774	[21, 24, 26, 29]
PSC	4	Fixed	1.421 (1.043–1.936)	**0.026**	[21, 24, 26, 29]
MT	3	Random	1.209 (0.663–2.204)	0.535	[24, 26, 29]
*GSTM1* null + *GSTT1* null:	6	Fixed	1.069 (0.843–1.356)	0.581	[22, 24, 26, 27, 29, 30]
*GSTM1* positive + *GSTT1* positive:	6	Random	1.005 (0.658–1.536)	0.981	[22, 24, 26, 27, 29, 30]

*GSTM1* positive + *GSTT1* null:	5	Random	1.281 (0.840–1.954)	0.250	[22, 24, 27, 29, 30]

*GSTM1*: glutathione S-transferase M1; *GSTT1*: glutathione S-transferase T1. CC: cortical cataract; NC: nuclear cataract; PSC: posterior subcapsular cataract; MT: mixed type cataract. Fixed: a fixed effects model (Mantel-Haenszel method); Random: the random effects model (DerSimonian-Laird method).
